# Enhanced Benefit of STA-MCA Bypass Surgery in Chronic Terminal Internal Carotid and/or Middle Cerebral Artery Occlusion Patients With Impaired Collateral Circulation: Introducing a Novel Assessment Approach for Collateral Compensation

**DOI:** 10.1155/emmi/5059097

**Published:** 2025-01-16

**Authors:** Cheng Qiu, Yanping Zhang, Zhiqiang Yu, Yonghui Xu, Yongjiang Huang, Tianci Huang, Jun Ma, Jinbing Zhao

**Affiliations:** ^1^Nanjing Comprehensive Stroke Center, Affiliated Nanjing Brain Hospital of Nanjing Medical University, Nanjing, China; ^2^Neurosurgery Department, Affiliated Hospital of Jining Medical University, Jining, China

**Keywords:** early-arriving flow proportion, middle cerebral artery occlusion, superficial temporal artery to middle cerebral artery bypass surgery, terminal internal carotid artery

## Abstract

**Background:** Ischemic stroke is one of the major emergency diseases leading to death and disability worldwide, characterized by its acute onset and the urgent need for prompt medical intervention to reduce mortality and long-term disability. Chronic terminal internal carotid artery and/or middle cerebral artery occlusion (CTI/MCAO) is an important subtype of intracranial artery occlusive disease. The superficial temporal artery-to-MCA (STA-MCA) bypass has been proposed to improve cerebral blood flow (CBF) and cerebrovascular reserve (CVR), potentially enhancing neurological outcomes. However, its safety and efficacy in CTI/MCAO patients remain controversial.

**Methods:** A total of 107 CTI/MCAO patients from Nanjing Brain Hospital, enrolled between July 2019 and June 2022, were divided into surgical and medical treatment groups. Cerebral perfusion and collateral formation were evaluated using pseudocontinuous arterial spin labeling (pCASL) and digital subtraction angiography (DSA). Modified Rankin scale (mRS) score and complication rates were compared between the two groups. In addition, correlations between Matsushima grades, early-arriving flow proportion (EFP), and lesion-side cerebrovascular (LCBV) scores were analyzed.

**Results:** The surgical group showed significantly lower mRS scores than the medical group (*p*=0.018), with no significant differences in complication rates at the 6-month follow-up (*p*=0.861). CBF differed significantly among affected MCA segments (*p* < 0.001), particularly in the insular and opercular regions (M2-M3) (*p*=0.006). Matsushima grades in unilateral CTI/MCAO patients were negatively correlated with preoperative LCBV scores (*γ*_s_ = −0.468, *p*=0.005) and EFP (*γ*_s_ = −0.648, *p*=0.007). EFP demonstrated high accuracy in predicting LCBV scores in CTI/MCAO patients (AUC = 0.902, *p*=0.004).

**Conclusion:** STA-MCA bypass surgery improved neurological outcomes in CTI/MCAO patients, particularly those with poor preoperative collateral compensation. EFP may serve as a reliable, noninvasive tool for assessing collateral circulation status in this population.

## 1. Introduction

Ischemic stroke is one of the major emergency diseases leading death and disability worldwide, characterized by its acute onset and the urgent need for prompt medical intervention to reduce mortality and long-term disability [1, 2]. In Western countries, predominantly among White populations, cardioembolism accounts for the major cause of ischemic stroke (28%), while in Asian populations, intracranial artery stenosis/occlusion (ICASO) stands as the primary cause of ischemic stroke [3].

Chronic terminal internal carotid artery and/or middle cerebral artery occlusion (CTI/MCAO) is a significant type of intracranial artery occlusive disease, often attributed to atherosclerosis. In patients with CTI/MCAO, the brain enters a decompensated state of ischemia and hypoxia, leading to the development of functional collateral circulation in some patients [4]. Despite medical treatment, CTI/MCAO remains an important cause of ischemic attack (TIA), neurological dysfunction, and stroke in considerable proportion of patients. Therefore, surgical treatment, such as the superficial temporal artery to MCA (STA-MCA) bypass, are proposed to enhance cerebral blood flow (CBF) and cerebrovascular reserve (CVR), offering potential benefits for improving neurological functions [5].

STA-MCA bypass surgery, first performed by Professor Yasargil in 1969, has remained a subject of debate regarding its ability to reduce stroke risk in patients with ischemic cerebrovascular disease. However, recent advancements in surgical techniques and evaluation methods have demonstrated that, when accompanied by systematic preoperative assessment and stringent perioperative management, STA-MCA bypass surgery can significantly reduce the long-term risk of stroke in these patients [6–8]. Therefore, this study aims to assess the benefits of STA-MCA bypass surgery in CTI/MCAO patients compared to a medicine treatment group.

## 2. Methods

### 2.1. Study Participants

A total of 107 patients with unilateral CTI/MCAO were continuously enrolled in the study: 58 received surgical treatment, while 49 received medical treatment. The inclusion criteria were as follows: (1) Unilateral CTI/MCAO confirmed by digital subtraction angiography (DSA), with any additional cerebral artery stenosis not exceeding 50%. (2) Confirmation of CBF reduction and hemodynamic disturbances in unilateral CTI/MCAO patients through arterial spin labeling (ASL) or CT perfusion (CTP). (3) Patients with acute cerebral infarction who had received medical treatment for at least 1 month, with follow-up MR scans showing no new infarctions, or had remained stable for approximately 2 months after medical treatment. (4) A modified Rankin scale (mRS) score ≤ 2. (5) Patients and their relatives consented to participate in the study by signing informed consent forms. In addition, symptoms and timing of onset are not stated in the entry criteria for the surgical group.

Exclusion criteria included the following: (1) intracranial stenosis ≥ 50% or confirmed moyamoya syndrome via DSA; (2) no definite CBF decrease observed on ASL or CTP; (3) severe underlying diseases or significant neurological dysfunction, defined as an mRS score > 3; (4) patients or their relatives declined participation. The patient flow diagram is presented in Supporting Figure 1.

This study was approved by the Ethics Committee of Nanjing Brain Hospital.

### 2.2. Clinical Evaluation

All 107 patients underwent DSA to evaluate cerebral arteries occlusion and collateral compensation. The degree of vascular occlusion was evaluated using modified thrombolysis in cerebral infarction (mTICI) with the following categories: 0 (no reperfusion), 1 (minimal flow past the occluded site but no perfusion), 2a (minor partial perfusion), 2b (major partial reperfusion), and 3 (complete perfusion) [9]. Collateral circulation was assessed by the American Society of Intervention and Therapeutic Neuroradiology/Society of Interventional Radiology (ASITN/SIR), categorized as 0 (no collaterals visible), 1 (sluggish collateral flow to the ischemic periphery with persistent perfusion defects), 2 (rapid collateral flow to the ischemic periphery with persistent perfusion defects), 3 (sluggish but complete flow to the ischemic territory in the late venous period), and 4 (complete or rapid collaterals to entire ischemic territory) [10]. The scores from both grading systems were combined into a single total score, termed the lesion-side cerebrovascular score (LCBV score).

Patients underwent a follow-up DSA 6 months after treatment. Collateral compensation after bypass surgery was evaluated according to the Matsushima grading system, with the following categories: 0 (no neovascularization in the target MCA territory), 1 (neovascularization presents in less than 1/3 of the MCA territory), 2 (between 1/3 and 2/3), and 3 (more than 2/3) [11].

All patients underwent ASL or CTP to quantify the CBF. ASL data were obtained by pseudocontinuous ASL (pCASL) sequences from a 3.0TMR scanner (Discovery MR750. General Electric Healthcare, USA). Each raw ASL scan had a 3-dimensional fast gradient and spin-echo readout module with background suppression. Besides, we applied multiple postlabeling delays (PLDs) of pCASL scans for more accurate evaluation of cerebral perfusion. The standard and long PLDs were set as 1.525 and 2.525 s. Postprocessing of the data was performed with MATLAB. WFU Pick Atlas tools (http://www.nitrc.org/projects/wfu_pickatlas) were used to create masks for 4 regions of interest (ROIs), including all MCA territory, the horizontal segment of the MCA (M1), the insula and operculum segments of the MCA (M2-M3), the cortical segment of the MCA (M4-M5) [12, 13]. Using MRIcron software to quantify CBF between different ROIs and calculate ΔCBF (ΔCBF = CBF_1. 525s_ of the contralateral side/CBF_1.525 s_ s of the lesion side). Early-arriving flow proportion (EFP) was defined as CBF_1.525s_ of lesion side/CBF_2.525s_ of the contralateral side [14].

The mRS was used to evaluate neurological function at the time of admission, discharge, and during the follow-up period. Patients who underwent the surgical treatment received postoperative CT and CT angiography (CTA) to check the patency of bypass arteries and the presence of infarcts or hemorrhage.

### 2.3. Surgical Treatment

The bypass surgery was performed under general anesthesia. The patients were positioned supine, and an arc-shaped or horseshoe-shaped incision was made in the ipsilateral frontotemporal region. The frontal or parietal branch of the STA was isolated as the donor artery and flushed with heparinized saline solution. The donor artery was trimmed to an adequate length, and its terminal end was stained with methylene blue and soaked in papaverine to prevent vasospasm. The M4 branches were identified using intraoperative indocyanine green angiography and preoperative cerebral perfusion assessment. An oval-shaped incision was made along the recipient M4 branch. An end to side anastomosis of the donor STA to the recipient MCA was performed with 10–12 interrupted stitches by 10-0 nylon sutures. Postanastomosis completion, indocyanine green angiography and Flow 800 were performed to assess the patency and flow characteristics. The incision was closed after adequate hemostasis.

### 2.4. Medical Treatment

Upon diagnosis of CTI/MCAO by DSA, medical treatment was initiated. Patients received double antiplatelet therapy and intensive statin therapy during the first 3 months. Commonly used medications included aspirin (100 mg/d), clopidogrel (75 mg/d), and atorvastatin (40 mg/d). Some patients' prescription were adjusted based on thrombelastography (TEG) and CYP2C19 gene examination, which involved changing the dose of clopidogrel or replacing it with ticagrelor and cilostazol. Subsequently, single-antiplatelet therapy and regular statin therapy would be continued after the 3-month intensive treatment.

### 2.5. Follow-Up

After enrollment, all patients underwent clinical and imaging follow-up. Clinical follow-up included outpatient visits, inpatient monitoring, and telephone assessments to assess improvements in initial symptoms, occurrence of new cerebrovascular events, posttreatment complications, and mRS scores. Imaging follow-up was conducted 3–6 months posttreatment, using DSA, CTA, CTP, and/or ASL to evaluate bypass patency and cerebral perfusion.

### 2.6. Statistical Analysis

Data following a normal distribution were presented as mean ± standard deviation, with comparisons between groups conducted using the *t*-test and multiple group comparisons performed using one-way ANOVA. For data that did not follow a normal distribution, median and quartile values (P50, [P25, P75]) are presented, and between-group comparisons were made using the rank-sum test. Categorical data are expressed as frequencies and percentages, with group comparisons analyzed using the Chi-square test. Receiver operating characteristic (ROC) curve analysis was employed for continuous quantitative variables, with optimal cutoff values determined based on the Youden index criterion. Statistical analyses were performed using SPSS 26.0 (IBM Incorporation, Chicago, USA) and GraphPad Prism 8.0.1 (GraphPad Software, San Diego, USA). A *p* value of < 0.05 was considered statistically significant.

## 3. Results

### 3.1. Characteristics of Enrolled Patients

Among the 107 enrolled patients, 58 received surgical treatment, while 49 received medical treatment (Table 1 and Supporting Table 1). There were no notable differences in terms of age, sex, stroke types, comorbidities, or mRS scores. Blood biochemical examination showed no significant differences between the two groups.

A total of 51 patients in the surgical treatment group and 43 patients in the medical treatment group completed clinical and imaging follow-up (Table 2). In the surgical group, all patients underwent CT, CTA, and MRI examinations to evaluate the presence of new infarcts or hemorrhages as well as the patency of bypasses. On the first postoperative day, CTA revealed patent bypass vessels in all cases. One patient developed a new ipsilateral intracerebral hemorrhage on the first day after surgery, while another developed a new ipsilateral infarction on the fifth day following surgery. Temporal neurological dysfunction, including aphasia and decreased limb muscle strength, was observed in six patients. Seizures occurred in four patients. No severe complications were reported during hospitalization in the medical treatment group.

### 3.2. Neurological Dysfunctions Improved in Surgical Treatment Group

The preoperative mRS scores of 58 patients in the surgical treatment group were distributed as follows: 0 in 4 patients, 1 in 24 patients, and 2 in 30 patients. In comparison, among the 49 patients in the medical treatment group, 4 patients had an mRS score of 0, while the remaining had scores of 1 (19 patients) or 2 (26 patients). Notably, no significant difference was observed in the pretreatment mRS scores between these two groups.

Following treatment, 51 patients in the surgical treatment group were followed up. Among them, 20 patients achieved an mRS score of 0, 23 patients scored 1, and 7 patients scored 2. Only one patient received a score of 4. In the medical treatment group, 43 patients were followed-up, with 6 achieving an mRS score of 0, 27 scoring 1, 9 scoring 2, and 1 patient scoring 3.

**Figure 1:**
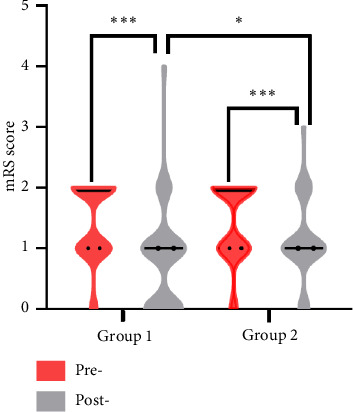
mRS score between different groups. Group 1: surgical treatment group; Group 2: medical treatment group. ⁣^∗^*p* < 0.05, ⁣^∗∗^*p* < 0.01, and ⁣^∗∗∗^*p* < 0.001. Pre-: pretreatment; post-: posttreatment.

As shown in Figure 1, after treatment, patients in the surgical treatment group exhibited statistically significant reduction in mRS scores (*p* < 0.001). A similar trend was observed in the medical treatment group (*p*=0.009) (Table 3). Furthermore, there was a statistically significant difference in posttreatment mRS scores between these two groups (*p*=0.018) (Table 2).

At the 6-month follow-up, among the 51 patients in the surgical treatment group, 4 patients experienced postoperative complications, including one case of cerebral hemorrhage and two cases of ipsilateral cerebral infarction, as well as three cases of TIA. Excluding stroke events that occurred during hospitalization, there was only one new case of cerebral infarction and three cases of TIA observed during the 6-month follow-up period. In the medical treatment group, two out of 43 patients experienced a cerebral hemorrhage at the lesion site, while one patient had a cerebral infarction and five patients suffered from TIA. No significant difference in complication incidence was observed between the two groups (Table 2).

### 3.3. CBF of Different Segments of MCA Assessed Using pCASL

**Figure 2:**
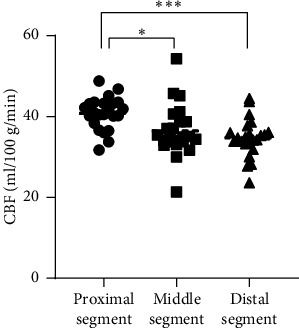
CBF of different MCA regions prior to treatment (PLD = 1.525 s). Proximal segment: horizontal segment of the MCA; middle segment: insula and operculum segment of the MCA; distal segment: cortical segment of the MCA. ⁣^∗^*p* < 0.05, ⁣^∗∗^*p* < 0.01, and ⁣^∗∗∗^*p* < 0.001.

**Figure 3:**
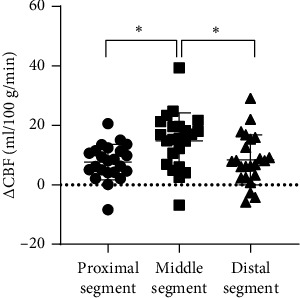
ΔCBF of different MCA regions prior to treatment (PLD = 1.525 s). Proximal segment: horizontal segment of the MCA; middle segment: insula and operculum segment of the MCA; distal segment: cortical segment of the MCA. ⁣^∗^*p* < 0.05, ⁣^∗∗^*p* < 0.01, and ⁣^∗∗∗^*p* < 0.001. ΔCBF = CBF 1.525 s of the contralateral side/CBF 1.525 s of the lesion side.

Before treatment, a two-phase ASL examination was conducted on 23 CTI/MCAO patients. The values of CBF and ΔCBF for an ipsilateral hemisphere are presented in Table 4. Statistically significant differences were observed between the CBF_1_ and CBF_2_ (*p*=0.008), as well as between CBF_1_ and CBF_3_ (*p* < 0.001). However, no significant difference was found between CBF_2_ and CBF_3_ (*p*=0.297), as shown in Figure 2. ΔCBF_1_ and ΔCBF_2_ exhibited significant differences (*p*=0.004), as did ΔCBF_2_ and ΔCBF_3_ (*p*=0.02). However, no significant difference was observed between the ΔCBF1 and ΔCBF3 (*p*=0.717) (Figure 3).

### 3.4. Patients With Inadequate Collateral Compensation Could Benefit More From STA-MCA Bypass Surgery

In the surgical treatment group, 35 patients underwent a follow-up DSA examination at 6 months postoperation. The efficacy of the bypass surgery was assessed using the Matsushima grading system, which revealed the following distribution of grades: 6 patients with grade 0, 14 with grade 1, 9 with grade 2, and 6 with grade 3.

**Figure 4:**
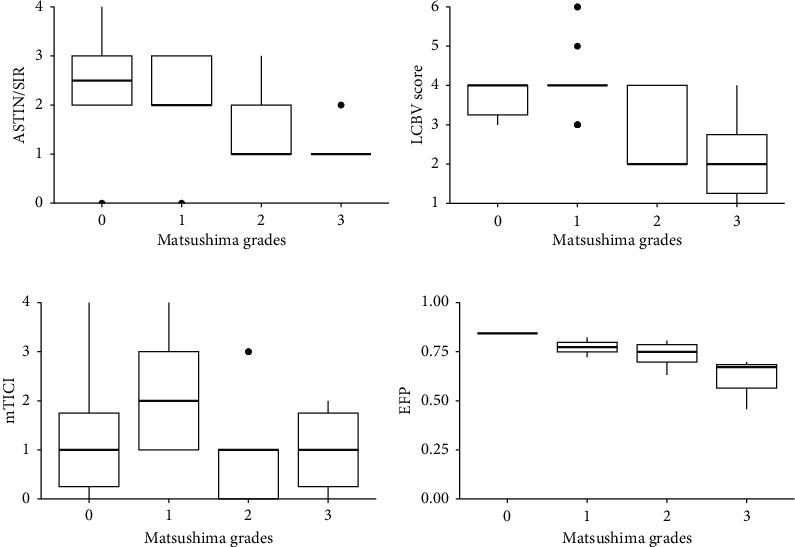
Correlation analysis among Matsushima grades with ASITN/SIR score, LCBV score, mTICI score, and EFP. ASITN/SIR score: the American Society of Intervention and Therapeutic Neuroradiology/Society of Interventional Radiology; mTICI: modified thrombolysis in cerebral infarction; LCBV score: the lesion side cerebrovascular score; EFP: early-arriving flow proportion.

Preoperative mTICI and ASITN/SIR scores for these 35 patients are presented in Figure 4. The LCBV score, derived from a combination of ASITN/SIR and mTICI score, is also shown in Figure 4 (Figure 4). The Spearman correlation analysis revealed significant negative associations between Matsushima grades and ASITN/SIR score (*γ*_s_ = −0.379, *p*=0.025), as well as LCBV score (*γ*_s_ = −0.468, *p*=0.005) (Figures 4(a) and 4(b)). However, no significant correlation was found between Matsushima grades and mTICI score (Figure 4(c)).

### 3.5. EFP Could Assess the Status of Collateral Circulation in CTI/MCAO Patients Noninvasively

A two-phase ASL examination was performed on 23 CTI/MCAO patients prior to treatment. Of these, 16 patients underwent surgical treatment and DSA follow-up. The Spearman correlation analysis revealed significant negative associations between Matsushima grades and preoperative EFP (*γ*_s_ = −0.648, *p*=0.007) (Figure 4(d)).

**Figure 5:**
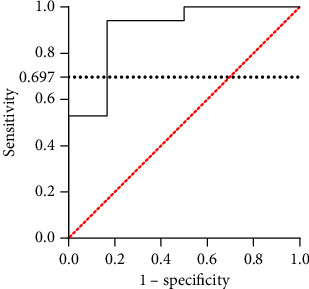
The receiver operating characteristic (ROC) curve of EFP. AUC = 0.902, *p*=0.004, Youden index = 0.775, and cut off = 0.697.

The EFP and LCBV scores were used to construct an ROC curve, with an LCBV score < 3 defined as indicative of inadequate CBF. The analysis showed a significant area under the curve (AUC) of 0.902, with a *p* value of 0.004. Maximizing the Youden index yielded an EFP value of 0.697 (Figure 5).

## 4. Discussion

Despite the continuous effort in neurointerventional techniques and materials, CTI/MCAO remains a significant cause of ischemic stroke (including recurrent stroke). As a major emergency disease, stroke can result in acute blood supply loss to the brain tissue and subsequent irreversible ischemic damage. Due to the narrow time window for stroke treatment, comprehensive management of stroke requires joint efforts in public education, the emergency transport system, medical/surgery treatment approaches, and, importantly, disease prevention. CTI/MCAO has been recognized as an important risk factor of ischemic strokes [15, 16], and it stands as a debilitating problem affecting about 45% of stroke patients [3, 17, 18]. Even receiving drug-based medical treatment (dual antiplatelet and lipid-lowering), most patients still experience occluded vessels and at a high risk of recurrent acute stroke [18]. Therefore, further exploration on the treatment of CTI/MCAO is essential to improve cerebral artery blood flow, reduce the onset of (recurrent) acute cerebrovascular disease, and prevent long-term mortality and disability.

Although cerebral revascularization has been proposed as a crucial therapeutic approach for acute occlusive cerebrovascular disease, there is a lack of consensus on the therapeutic effect of CTI/MCAO patients to prevent further emergency diseases and improve outcomes. The International EC-IC Bypass Study failed to demonstrate that EC-IC bypass surgery reduces the recurrence of ischemic stroke in CTI/MCAO patients compared to medical treatment [19]. Interestingly, the Carotid Occlusion Surgery Study (COSS) and the Japanese EC-IC Bypass Trial (JET) study yielded opposing results [20]. In the present study, we analyzed 107 adult patients who met the criteria for unilateral CTI/MCAO. The complication rates did not differ significantly between the surgical and the medical treatment group during the 6-month follow-up period, which may be considered relatively short. However, previous studies have showed that, over a longer follow-up period, the incidence of posttreatment complications was lower in the surgical treatment group compared to the medical treatment group [7, 8]. Moreover, our study demonstrated that bypass surgery significantly improved neurological function in patients without increasing stroke incidence.

Among the 23 patients who underwent two-phase pCASL tests, we observed a reduction in CBF within the affected MCA territory compared to the contralateral side. This reduction was most prominent in the insula and operculum segments of MCA (M2-M3), likely due to the influence of collateral circulation. In unilateral CTI/MCAO patients, collaterals originating from the occluded ICA predominantly supply the M1 segment, while blood flow in M4-5 segments mainly consisted of reverse flow from distal ACA branches. We hypothesized that this specific pattern of collateral circulation contributes to more severe ischemia in the insula and operculum segments of MCA (M2-M3). A deeper bypass surgery (e.g., STA-M2 bypass) may therefore provide enhanced blood perfusion for unilateral CTI/MCAO patients.

A study involving 31 patients with unilateral CTI/MCAO demonstrated that the internal maxillary artery (IMA)—radial artery graft (RAG)—MCA bypass surgery could enhance cerebral perfusion and cerebral glucose metabolism, alleviate clinical symptoms, improve neurological function, and did not result in any new neurological deficits during the follow-up period of 2.19 ± 1.59 years [21]. This surgical approach may be particularly advantageous for patients with segmental occlusion and a well-functioning distal vascular bed. Moreover, some researchers have utilized the STA-MCA double anastomoses to augment blood flow further and achieve superior outcomes [22, 23]. In this present investigation, one patient underwent STA-MCA double anastomoses surgery; postoperative DSA examination revealed that the donor artery supplied more than two-thirds of the MCA area (Matsushima Grade 3). The patient experienced significant improvement in language function after surgery without encountering any unforeseen complications during the 3-year follow-up period. However, additional studies are still necessary to validate the safety and efficacy of these techniques.

Currently, there is no consensus on how to assess collateral circulation in unilateral CTI/MCAO patients. In the present study, mTICI score was used to evaluate occlusion, the ASITN/SIR score assessed collateral compensation, and the combination of these two scores was used to evaluate LCBV score. Our results showed that the therapeutic outcome of surgery in unilateral CTI/MCAO patients was negatively correlated with preoperative LCBV score. Specifically, patients with poorer preoperative cerebrovascular compensation showed more efficient revascularization after bypass artery. In addition, there was a significant negative association between surgical outcome and ASITN/SIR score, but no statistically significant correlation was found between surgical outcome and the mTICI score.

Among the 35 patients who underwent postoperative DSA examination, 4 cases showed STA-MCA bypass shrinkage, and 2 cases showed bypass artery patency without compensation to the MCA territory. Preoperative DSA indicated that in four of these cases, the ACA had compensated well for the MCA territory, while the remaining 2 cases had partial M1 segment occlusion with a well-preserved distal vessel bed. We hypothesize that this might be due to a conflict between the donor artery blood flow and intrinsic blood flow. When the blood flow from the donor artery counteracts the intrinsic blood flow, it can lead to cerebral ischemia, watershed infarction, overperfusion, or even cerebral hemorrhage. Severe blood flow conflict would also deplete the hemodynamic potential of donor artery, resulting in long-term stenosis or occlusion [24]. Therefore, we maintain anterograde blood flow in the donor artery in alignment with the dominant intrinsic blood flow after bypass surgery. To avoid blood flow conflict, we recommended adjusting the direction of blood flow by modifying stitch margin.

Moreover, we also found that there was a significant negative association between surgical outcome and preoperative EFP. In other words, patients with lower preoperative EFP showed more extensive revascularization after bypassing artery, which was consistent with the LCBV score. EFP was the ratio of blood flow arrival time of the lesion side to the contralateral side. It could reflect the establishment of collateral circulation in unilateral CTI/MCAO patients. Previous studies had used oxygen extraction fraction as an indication of bypass surgery [25, 26], but the PET/CT examination was difficult to popularize in most hospitals. We applied pCASL examination for CBF evaluation, which was much more convenient with huge equipment investment. Therefore, we performed the ROC curve analysis to evaluate the predictive capability of EFP. We found that EFP had high accuracy in predicting the anterior circulation compensation of unilateral CTI/MCAO patients, and the cut-off value of EFP was set at 0.697. The EFP could be used as an indicator to quantitatively predict the anterior circulation of unilateral CTI/MCAO patients.

## 5. Conclusion

STA-MCA bypass surgery improved neurological outcomes in patients with CTI/MCAO, particularly those with poor preoperative collateral compensation. EFP may serve as a reliable, noninvasive tool for assessing the collateral circulation status in these patients. Further studies are needed to confirm our results in multicenter trials with larger sample sizes and longer follow-up.

## Figures and Tables

**Table 1:** The clinical characteristics of enrolled unilateral CTI/MCAO patients prior to treatment.

	Cases (*n* = 107)	Surgical group (*n* = 58)	Medical group (*n* = 49)	*p*
Age (y)	56.52 ± 11.22	54.66 ± 10.68	58.73 ± 11.54	0.061
Sex (male, %)	56 (52.34%)	30 (51.72%)	26 (53.06%)	0.89
Type				
Infarction	53 (49.53%)	26 (44.83%)	27 (55.10%)	0.29
Hemorrhage	9 (8.41%)	5 (8.62%)	4 (8.16%)	1
Untypical	45 (42.06%)	27 (46.55%)	18 (36.73%)	0.305
Diabetes (yes, %)	25 (23.36%)	14 (24.14%)	11 (22.45%)	0.837
Hypertension (yes, %)	58 (54.21%)	28 (48.28%)	30 (61.22%)	0.18
mRS score	2 (1, 2)	2 (1, 2)	2 (1, 2)	0.95

**Table 2:** The clinical characteristics of enrolled CTI/MCAO patients post-treatment.

	Cases (*n* = 94)	Surgical group (*n* = 51)	Medical group (*n* = 43)	*p*
mRS 6-month posttreatment	1 (0,1)	1 (0,1)	1 (1,2)	0.018
Stroke during hospitalization	2 (2.13%)	2 (3.92%)	0 (0%)	0.498
Complications within 6 months post-treatment				
Total	14 (14.89%)	6 (11.76%)	8 (18.6%)	0.861
Hemorrhage	3 (3.19%)	1 (1.96%)	2 (4.65%)	0.591
Infarction	3 (3.19%)	2 (3.92%)	1 (2.33%)	0.99
TIA	8 (8.51%)	3 (5.88%)	5 (11.63%)	0.463
Complications from 2 to 6 months posttreatment				
Total	12 (12.77%)	4 (7.84%)	8 (18.6%)	0.119
Hemorrhage	2 (2.13%)	0 (0%)	2 (4.65%)	0.207
Infarction	2 (2.13%)	1 (1.96%)	1 (2.33%)	0.99
TIA	8 (8.51%)	3 (5.88%)	5 (11.63%)	0.463

**Table 3:** The mRS score pre- and posttreatment.

	mRS score pretreatment	mRS score 6-m posttreatment	*p*
Surgical treatment	2 (1,2)	1 (0,1)	< 0.001
Medical treatment	2 (1,2)	1 (1,2)	0.009

**Table 4:** The CBF and ΔCBF (ml/100 g/min) in different MCA regions prior to treatment (PLD = 1.525 s).

	Horizontal segment	Insula and operculum segment	Cortical segment	*p*
CBF	40.81 ± 4.01	36.43 ± 6.33	34.69 ± 4.71	< 0.001
ΔCBF	7.63 ± 5.98	14.78 ± 9.40	8.42 ± 8.42	0.006

## Data Availability

All the data included in this study are available by contacting the corresponding authors.

## Supporting Information

Supporting Information 1 Supporting Figure 1: CONSORT 2010 flow diagram.

Supporting Information 2 Supporting Table 1: Blood biochemical examination of enrolled participants.
