# Supplementary figures and images for: Enhanced Benefit of STA-MCA Bypass Surgery in Chronic Terminal Internal Carotid and/or Middle Cerebral Artery Occlusion Patients With Impaired Collateral Circulation: Introducing a Novel Assessment Approach for Collateral Compensation
Source: Emerg Med Int. 2025 Jan 16;2025:5059097. doi: 10.1155/emmi/5059097 (PMC11756939; doi:10.1155/emmi/5059097)

## CONSORT 2010 Flow Diagram

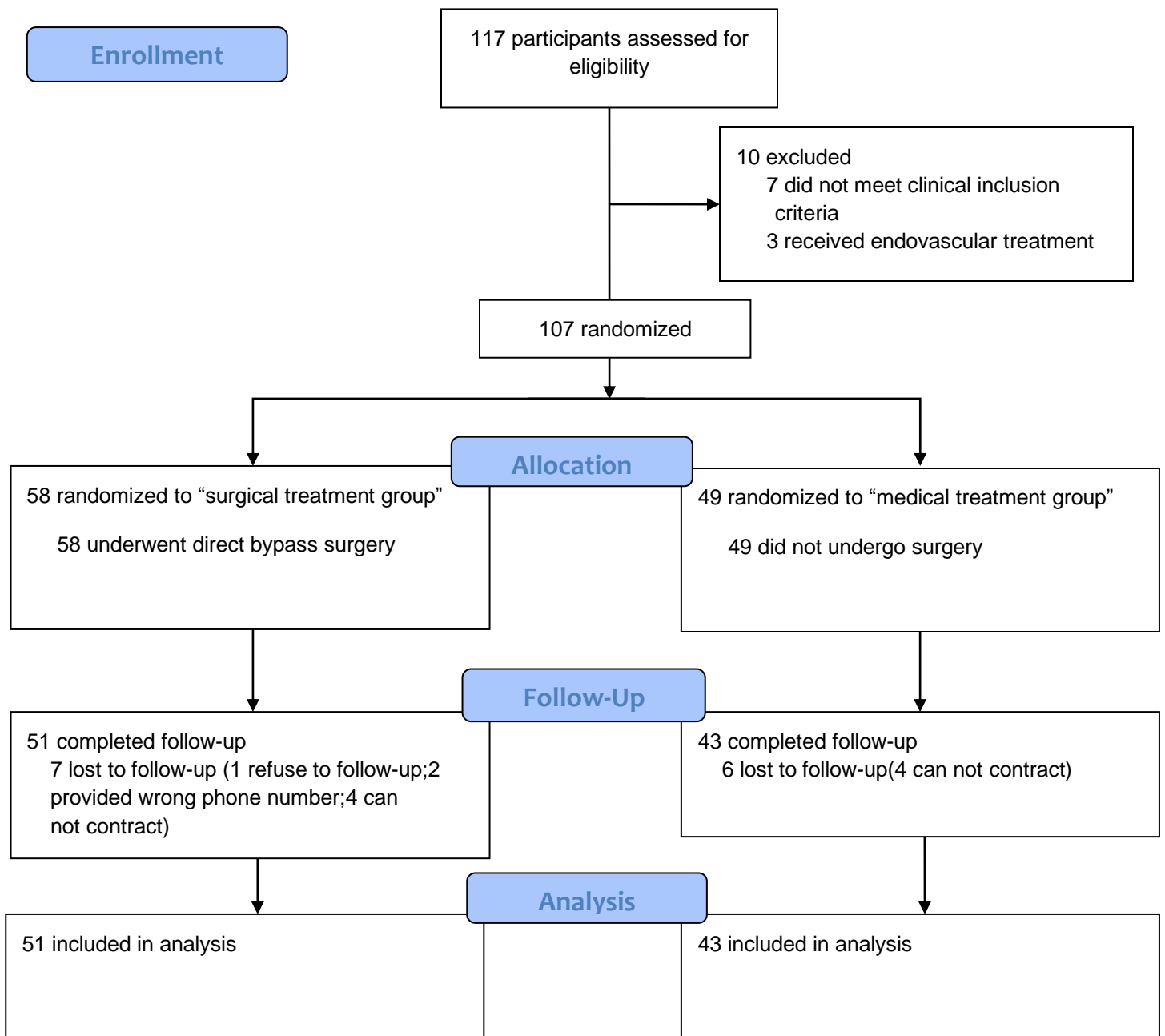

Supplement: Supporting Information 1 — Supporting Figure 1: CONSORT 2010 flow diagram. [file 5059097.f1.pdf]
