# Supplementary material for: Enhanced Benefit of STA-MCA Bypass Surgery in Chronic Terminal Internal Carotid and/or Middle Cerebral Artery Occlusion Patients With Impaired Collateral Circulation: Introducing a Novel Assessment Approach for Collateral Compensation
Source: Emerg Med Int. 2025 Jan 16;2025:5059097. doi: 10.1155/emmi/5059097 (PMC11756939; doi:10.1155/emmi/5059097)
Supplement: Supporting Information 2 — Supporting Table 1: Blood biochemical examination of enrolled participants. [file 5059097.f2.docx]

**Supplement Table 1.** Blood biochemical examination of enrolled participants

|  | Total (N=107) |
| --- | --- |
| WBC | 6.2 (1.8) |
| RBC | 4.5 (0.5) |
| HB | 132.9 (15.4) |
| HCT | 40.4 (4.0) |
| MCV | 89.3 (6.6) |
| MCH | 29.4 (2.6) |
| MCHC | 329.1 (11.4) |
| RDWSD | 41.0 (3.7) |
| RDWCV | 13.0 (1.2) |
| PLT | 206.8 (60.0) |
| PT | 11.4 (0.6) |
| APTT | 32.4 (5.0) |
| Fibrinogen | 2.6 (0.6) |
| ALT | 25.7 (19.6) |
| AST | 19.8 (11.3) |
| Total Protein | 66.8 (4.5) |
| Glucose | 5.6 (2.3) |
